# Longitudinal Association between Physical Activity, Blood Lipids, and Risk of Dyslipidemia among Chinese Adults: Findings from the China Health and Nutrition Surveys in 2009 and 2015

**DOI:** 10.3390/nu15020341

**Published:** 2023-01-10

**Authors:** Qinpei Zou, Chang Su, Wenwen Du, Huijun Wang, Bing Zhang, Shuquan Luo, Tao Tan, Xiaoyun Song, Xiaoni Zhong, Huadong Zhang, Yaokai Chen

**Affiliations:** 1 Department of Infectious Diseases, Chongqing Public Health Medical Center, Chongqing 400036, China; 2 Chongqing Center for Disease Control and Prevention, Chongqing 400042, China; 3 School of Public Health, Research Center for Medicine and Social Development, Chongqing Medical University, Chongqing 400016, China; 4 National Institute for Nutrition and Health, Chinese Center for Disease Control and Prevention, Beijing 100050, China; 5 Chongqing Health Statistics Information Center, Chongqing 401120, China; 6 Dalian Center for Disease Control and Prevention, Dalian 116035, China

**Keywords:** physical activity, blood lipids, dyslipidemia

## Abstract

Physical activity is needed in today’s highly sedentary society. Research into Chinese trends with respect to the longitudinal association between changes in physical activity (PA) and dyslipidemia (DL) is limited. The present study assessed PA and PA-related changes associated with the level of serum lipids, and the risk of dyslipidemia stratified by gender, via a population-based longitudinal observational study among adults (>18 years old) residing in nine provinces in China. Data of 3380 adult participants, gleaned from two surveys in 2009 and 2015 used by the China Health and Nutrition Survey (CHNS), were analyzed. Three-level random intercept growth models were used to calculate the associations between total physical activity (TPA), the four activity subtypes, and changes in the level of blood lipids. Three-level logistic regression models were utilized to assess the association between TPA and the risk of DL. The highest level of TPA was observed to correlate with the lowest prevalence of DL. Compared with the lowest level of TPA, the highest level of TPA increases the concentration of HDL-C (β = 0.050, 95% CI = 0.005, 0.096) in males, and also reduces the risk in males of having low HDL-C by 31% (OR = 0.69, 95% CI = 0.52, 0.91). Occupational PA was positively associated with higher HDL-C levels in both genders (males: β = 0.004, 95% CI = 0.002, 0.007; females: β = 0.002, 95% CI = 0.000, 0.004), and leisure-time physical activity (LTPA) was negatively associated with TG levels in males. Increasing TPA benefited HDL-C levels in males, irrespective of the level of basic TPA of individuals. Physical activity (especially occupational PA) was superior in positively influencing HDL-C in males, but not in females. Leisure-time physical activity needs to be increased substantially in order to increase TPA to levels that would be beneficial to blood lipid levels and DL, irrespective of baseline TPA levels.

## 1. Introduction

Dyslipidemia (DL), which is characterized by high low-density lipoprotein cholesterol (LDL-C), low high-density lipoprotein cholesterol (HDL-C), high total cholesterol (TC), and high triglycerides (TG), has emerged as the second leading factor associated with the risk of cardiovascular disease (CVD), and has been reported to have caused an increase in the annual total mortality directly related to dyslipidemia in China from 2.51 million in 1990 to 3.97 million in 2016 [1,2]. Dyslipidemia has been recognized as an independent risk factor for atherosclerosis, coronary heart disease, and stroke, and is playing a vital role in the emerging epidemic of CVD in China. The latest report on Chinese nutrition and chronic diseases (2022) showed that the prevalence of DL among Chinese aged 18 years old and above was 35.6%. Additionally, the prevalence of hypercholesterolemia, hypertriglyceridemia, low HDL-C, and high LDL-C was 8.2%, 18.4%, 20.9%, and 8.0%, respectively, and the prevalence of DL rapidly increases as a function of age [3]. The analysis of the data from the China Patient-Centered Evaluative Assessment of Cardiac Events study reported a similar DL prevalence (33.80%) [2]. Serum lipids among Chinese adults during the three national cross-sectional surveys undertaken between 2002 and 2015 have increased sequentially [4]; in contrast, the USA experienced an improving trend in lipid levels between 1998 and 2010 [5].

Physical activity (PA) refers to all movement, and is classed into four types, including occupational PA, travel PA, domestic PA, and leisure-time PA (LTPA), and has been shown to contribute to the prevention and management of CVD [6]. Together with unprecedented global economic and social development, a host of lifestyle factors have altered daily life for everyone. Current global estimates indicate that 27.50% of adults and 81.00% of adolescents do not meet the global recommendations for PA set by the World Health Organization (WHO) [7]. In some countries, levels of inactivity can be as high as 70%, due to changing patterns of transportation, the increased use of technology, and urbanization [8]. A sedentary lifestyle with a lack of PA is the commonest secondary cause of dyslipidemia in developed nations [9]. Total physical activity (TPA) has declined sharply as urbanization has dramatically increased in China [10]. The global cost of physical inactivity in 2013 was estimated to be USD 54 billion per year in direct healthcare costs, with an additional cost of USD 14 billion attributable to lost productivity [8].

Previous studies have suggested that exercise has beneficial effects on serum lipid metabolism in healthy individuals and also in those with chronic illnesses [11]. No matter which form of activity is chosen, i.e., aerobic exercises, strength training, or high-intensity interval training, and whether long-term or short-term, PA promotes lipid metabolism by increasing HDL-C, and decreasing LDL-C, TC, and TG [12]. However, the concept of PA is different from exercise, which includes a broad range of daily activities [13]. When PA was assessed by questionnaire, O’Donovan, in a pooled analysis of nine cohorts, found that HDL-C could be improved by achieving the amount of LTPA recommended by PA guidelines for healthy individuals and those with low HDL-C [14]. Zhang analyzed the relationship between LTPA and blood lipids among 17,535 Chinese people aged from 18 to 78 years old, and found that a lack of LTPA, and obesity, were both associated with an increase in TG levels and a decrease in HDL-C levels, while obesity and a lack of PA had compounding effects on the abnormalities seen in TG and HDL-C levels [15].

In addition, there are other limitations that have not been addressed in previous studies. For example, both Chinese and international studies have reported the significant negative association between exercise or LTPA, and deranged blood lipids and the risk of DL [12,16,17,18]. However, Chinese adults tend to have much less LTPA or even no LTPA at all compared with their counterparts in other countries, and therefore, the results of international studies concerning the association between LTPA and deranged blood lipids or the risk of DL may not be applicable in China. Furthermore, most past studies have been based on cross-sectional surveys, which are relatively limited in their ability to make robust causal inferences, indicating that clinical cohort studies are required, especially in developing countries such as China. Moreover, evidence has shown that in Chinese adults there is a declining trend for PA over time, and it remains unclear whether any decrease in TPA over extended periods has an impact on blood lipids.

To address these knowledge gaps, data from Chinese adults (age ≥ 18 years) obtained in 2009 and 2015 from the China Health and Nutrition Survey (CHNS) were used to examine the association between PA, the subgroups of PA, changes in PA, and serum lipids, stratified by gender. We also aimed to assess the effect of TPA on the risk of DL.

## 2. Materials and Methods

### 2.1. Study Population

The CHNS is an ongoing multipurpose large-scale longitudinal survey, with the aim of capturing sociological, economic, and demographic factors that influence health and nutritional status across the Chinese life span. Initiated in 1989, China has seen a total of 11 CHNS surveys (1989, 1991, 1993, 1997, 2000, 2004, 2006, 2009, 2011, 2015, and 2018) being conducted thus far. The original survey used a multistage, random cluster design in eight provinces, using a weighted sampling method to select a stratified probability sample. Specific individuals participated in the survey repeatedly at each round unless they were lost to follow-up. Further details regarding the CHNS are described and can be accessed at the following internet website: https://www.cpc.unc.edu/projects/china (accessed on 9 January 2023), and elsewhere [19].

**Figure 1:**
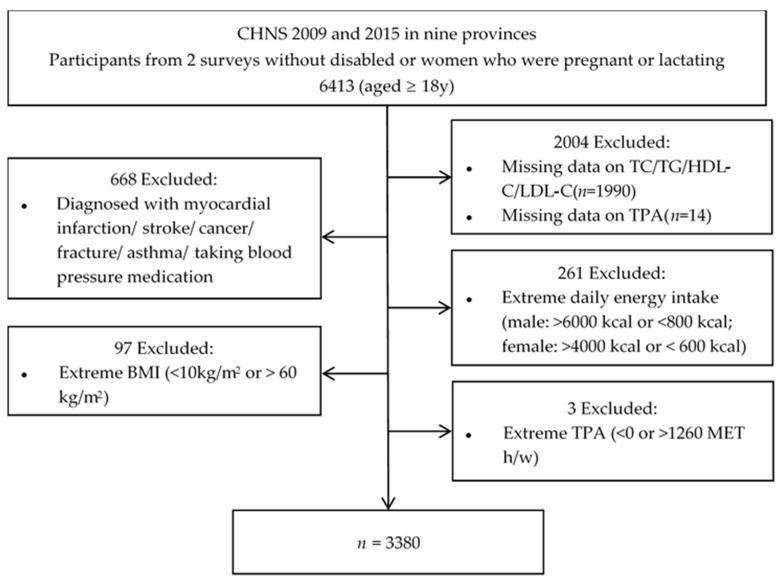
Flow chart of the included study participants from the China Health and Nutrition Survey (CHNS) in 2009 and 2015. TC, total cholesterol; TG, triglycerides; HDL-C, high-density lipoprotein cholesterol; LDL-C, low-density lipoprotein cholesterol; BMI, body mass index; TPA, total physical activity.

We used the data of the 2009 and 2015 surveys of the CHNS, because fasting blood sample results were available only for these specific surveys at the time of our analysis. Fifteen provinces were represented in 2015; however, only nine provinces were surveyed in 2009. In order to include only participants who had participated in both of these two surveys, the final number of provinces used was nine, and those provinces were Liaoning, Heilongjiang, Shandong, Jiangsu, Henan, Hunan, Hubei, Guizhou, and Guangxi. The 2009 survey included 4320 households within 216 communities (36 urban neighborhoods, 36 suburban neighborhoods, 36 towns, and 108 villages). The number of participants (age ≥ 18 years) recorded in the two surveys who were not disabled, not pregnant, and not lactating was 6413. Participants that omitted data on blood lipid profiles (*n* = 1990) or TPA (*n* = 14) were deleted. After excluding participants that were associated with myocardial infarction, stroke, cancer, fracture, asthma, or taking blood pressure medication (*n* = 668), and those associated with either extreme daily energy intake (higher than 6000 kcal/day or lower than 800 kcal/day for males, higher than 4000 kcal/day or lower than 600 kcal/day for females) (*n* = 261), those with extreme BMI (<10 kg/m^2^ or >60 kg/m^2^, *n* = 97), and those with extreme TPA (<0 or >1260 metabolic equivalents of task hours per week (MET·h/week), *n* = 3), a total of 3380 participants (6760 observations) were included in the final analysis (Figure 1).

The survey protocols, instruments, and process of obtaining informed consent for this study were approved by the National Institute for Nutrition and Health, the Chinese Center for Disease Control and Prevention (No. 201524-1), and the Institutional Review Committee of the University of North Carolina at Chapel Hill, and written informed consent was obtained from all the CHNS participants before data collection.

### 2.2. Physical Activity and Sedentary Assessment

We relied on a standard PA questionnaire (which has been used in this ongoing cohort study for almost 30 years [20]) to calculate the average MET hours per week to indicate PA level. This questionnaire included relevant questions on the intensity, frequency, and time spent on occupational, travel, leisure, and domestic movements (e.g., farming, biking, playing basketball, food preparation). The ratio of a person’s working metabolic rate relative to their resting (basal) metabolic rate was defined as a MET unit. Thus, the average MET hours per week measurements incorporated both the time spent on each activity and the average intensity of each activity (or sub-activity). We applied the appropriate estimated MET intensity values using the Compendium of Physical Activities, based on the lowest level of detail available for each time-use survey [21,22,23]. We categorized the total MET hours per week into four quartiles, i.e., Q1, Q2, Q3, and Q4, from the lowest to the highest PA level according to quartiles for each survey round, because there is currently no internationally recommended standardized categorization for PA. When we analyzed the PA subtype and serum lipids data, PA was used as a continuous variable, and its unit was set at 4.0 MET, which represents moderate PA. Details with respect to the calculation of these values have been previously described elsewhere [19,20].

The relationship between TPA and certain specific lipid changes seen after 6 years was observed to be statistically significant. In order to describe the association between this interaction and the serum lipids, we categorized TPA into low, moderate, and vigorous activity, according to tertiles in 2009. The change in value was defined as the TPA in 2015 minus the TPA in 2009. This result was not normally distributed, with no significant difference between males and females. Therefore, we stratified the TPA change value into tertiles, which were ‘decreased’ (−267.64~−31.22 MET MET·h/week), ‘stable’ (−31.19~0.41 MET MET·h/week), and ‘increased’ (0.46~259.00 MET·h/week). There were thus nine categories defining the basic TPA × change value, which included: vigorous and increased, vigorous and stable, vigorous and decreased, moderate and increased, moderate and stable, moderate and decreased, low and increased, low and stable, and low and decreased. Based on our data, the low and decreased group was not represented; we thus used the data of the other eight groups, and the last group was used as a reference group.

Sedentary behaviors were calculated as the average hours per day (hour/day) spent in various non-occupational recreational activities, including lying down, sitting (reading/writing, playing board games, and using a computer or other forms of screen entertainment), and watching TV or movies/videos. A summation of the time spent engaged in these activities was used to obtain the total time expenditure on sedentary behaviors, which was not included in the PA calculation.

### 2.3. Anthropometrics and Blood Biomarker Measurements

Trained health workers or nurses measured the weight, height, and waist circumference of participants, following standardized procedures. Height and weight were measured based on a standard protocol. Weight in lightweight clothing was measured to the nearest 0.1 kg, with the participant standing without shoes, and height was measured without shoes to the nearest 0.1 cm using Seca 206 wall-mounted metal tapes. We calculated BMI as weight in kg divided by height in square meters. Waist circumference was measured in centimeters at the mid-point between the lowest rib margin and the top of the iliac crest during light breathing using a Seca tape measure.

Overnight fasting blood was collected from the antecubital vein of participants in the morning. Plasma and serum samples were processed (centrifuged at 2000 g for 10 min at room temperature and separated into 9 aliquots) within two hours of collection in local hospitals. The aliquots were frozen and stored at −86 degrees centigrade for later laboratory analysis. All samples were analyzed in a national laboratory in Beijing with strict quality control. Serum TG, TC, HDL-C, and LDL-C were measured via the CHOD-PAP (Kyowa Medex Co., Ltd., Tokyo, Japan) method.

According to the 2016 Chinese Adult Dyslipidemia Prevention Guideline, the definition of DL is a TC ≥ 6.2 mmol/L (240 mg/dL) and/or a TG ≥ 2.3 mmol/L (200 mg/dL) and/or a HDL-C < 1.0 mmol/L (40 mg/dL) and/or a LDL-C ≥ 4.1 mmol/L (160 mg/dL). Four clinical subtypes of DL were identified. Hypercholesterolemia was defined as an isolated presence of a TC ≥ 6.2 mmol/L (240 mg/dL). Hypertriglyceridemia was defined as the isolated presence of TG ≥ 2.3 mmol/L (200 mg/dL). Combined hyperlipidemia was a combination of both hypercholesterolemia and hypertriglyceridemia. Low HDL-C was defined as the isolated presence of a HDL-C of <1.0 mmol/L (40 mg/dL).

### 2.4. Assessment of Covariates

Well-trained interviewers used standard questionnaires to collect sociodemographic data, including annual per capita household income, current smoking status, alcohol consumption, community information (urbanization index), and dietary intake. Educational level was divided into two categories, i.e., less than high school education and high school education and above. We defined marital status as “married” and “not married”. Participants reported their gross annual per capita household income according to household size, and their 2009 gross annual income was inflated to year 2015 equivalency. The community urbanization index, which was standardized and validated, captured the changes in 12 multidimensional components at the community level reflecting population density, economic activity, traditional markets, modern markets, transportation infrastructure, sanitation, communications, housing, education, diversity, health infrastructure, and social services [19,24]. We calculated the energy intake, energy from dietary fat, and dietary cholesterol, which were collected using three consecutive 24 h recalls (two weekdays and one weekend), in combination with the household weighing of condiments over the same three-day period, according to the Chinese Food Composition Tables.

### 2.5. Statistical Analysis

We calculated descriptive statistics for the individual demographic variables in 2009 and stratified them by gender. The median and the 25th and 75th percentiles were used to express continuous variables because of their abnormal distribution. Categorical variables were expressed as percentages. Kruskal–Wallis tests were used for continuous variables and Chi-square tests were conducted for categorical variables. Prevalence was defined as the ratio of all patients with DL for all participants in that year.

Unlike hypertension and diabetes, and apart from medication, DL as the dependent variable seemed to be more amenable to modification by diet and lifestyle improvement. A participant with DL is more likely to have normal blood lipids at the next survey round in the absence of medication if diet and lifestyle changes have been implemented. Similarly, TPA, as an independent variable, was utilized as the fixed variable in the statistical models in previous studies, because it was assumed that TPA was fixed. However, TPA should be treated as a volatile variable because it is easily changed as age increases and personal willingness to perform physical activities declines. Therefore, the multi-level random intercept growth model is more suitable for longitudinal data analysis than is the COX proportional hazards regression model because the latter model assumes that all variables are fixed.

Because of the nested data structure, the association between PA and blood lipids (or DL) may be presented as multiple-level relationships. HDL-C levels, LDL-C levels, TC levels, TG levels, and DL were our dependent variables. We tested the contribution made by the community level (third level), the individual level (second level), and the residual level (first level). The community level was found to contribute 5.18%, 7.06%, 6.92%, 3.70%, and 4.84% to HDL-C levels, LDL-C levels, TC levels, TG levels, and DL, respectively. All second level and third level contributions were calculated to be statistically significant (*p* < 0.01).

We used a three-level logistic regression model to assess associations between PA and the risk of DL. The model was adjusted for the specific survey round, baseline age, energy intake, energy from dietary fat, dietary cholesterol, BMI, waist circumference, whether currently smoking, whether an alcohol consumer, education, per capita household income, sedentary activity time, and the urbanization index. We then used a three-level random intercept growth model to assess the associations between PA and blood lipids. Three separate models were constructed. The main independent variable was PA, and this included TPA, occupational PA, travel PA, domestic PA, and LTPA, and the dependent variables were HDL-C levels, LDL-C levels, TC levels, and TG levels. We attempted to control as many confounding factors as possible. Model 1 was controlled for TPA, survey round, and baseline age. Based on Model 1, energy intake, energy from dietary fat, dietary cholesterol, BMI, waist circumference, current smoker, alcohol drinker, education, per capita household income, and sedentary activity time were included in Model 2. Model 3 additionally adjusted for the urbanization index, was based on Model 2. In all our models, the lowest quartile of TPA (Q1) was the reference level.

Additionally, for the four subtypes of PA, three-level random intercept growth models were constructed, which were adjusted for survey round, baseline age, energy intake, energy from dietary fat, dietary cholesterol, BMI, waist circumference, whether a current smoker, whether an alcohol consumer, education, per capita household income, sedentary activity time, and the urbanization index. We also used the three-level random intercept growth models to test the association between the baseline TPA level and its changing type within the six years under consideration (2009–2015) and blood lipids, adjusting for survey round, baseline age, energy intake, energy from dietary fat, dietary cholesterol, BMI, waist circumference, whether a current smoker, whether an alcohol consumer, and sedentary activity time.

All statistical tests and scatter diagrams were conducted using SAS software (version 9.4, SAS Institute Incorporated, Cary, NC, USA). The three-level random intercept growth model used the PROC MIXED process and the three-level logistic regression model used the PROC GLIMMIX process. All the continuous variables were centrally processed using (x − $\bar{x}$). Three-dimensional distribution graphs and forest plots were constructed using R software (version 3.6.2), utilizing the ggplot2 and forestplot packages, respectively. All *p*-values were two-sided and a *p*-value of <0.05 was considered to be statistically significant.

## 3. Results

### 3.1. Basic Characteristics of Participants in Each Survey Round

Table 1 shows the primary information of participants for each gender in 2009 and 2015. There were 3380 sample participants in 2009, and the number of participants in 2015 was identical. The percentage of male participants was 45.18%. The median baseline ages were 51.09 years in males and 49.62 years in females. The number of males with high school education and above was 26.59% in 2009, which was significantly higher than that of females (17.49%), and this prevalence increased for both genders in 2015. The annual per capita household income and the urbanization index between men and women did not significantly differ; however, these indices were statistically significantly higher in 2015 than in 2009. Smoking (56.65%) and alcohol consumption (63.85%) in males were statistically significantly higher than the prevalence in females in both survey rounds; however, the actual number of those who indulged in smoking and drinking declined in 2015. Energy intake (2390 kcal/day) and dietary cholesterol (243.36 mg/d) in 2009 were significantly higher in men compared to women (2000 kcal/d and 205.92 mg/d, respectively), and there was no difference in energy from dietary fat between men and women. A significant decrease in dietary variables was observed in 2015, compared to those in 2009. Body mass index and waist circumference increased as people aged. There was no statistical difference in BMI between genders, while waist circumference in males was significantly higher than in females. The median sedentary activity time in men (16 h) was also significantly higher than that in women (14 h), and there seemed to be a slight decline in sedentary activity time over the 6 years of the study period (Appendix A).

Median TPA decreased sharply during the period of the two surveys. Median TPA was 178.74 MET·h/week in 2009 and this decreased to 100.49 MET·h/week in 2015. The median TPA in females declined to a greater extent than that in males. The median TPAs for the quartile groups, from the lowest to the highest, were 40.78 MET·h/week, 126.52 MET·h/week, 245.12 MET·h/week, and 466.84 MET·h/week in 2009; however, TPA indices for each quartile fell precipitously in 2015. With respect to the four subtypes of PA, occupational PA accounted for the largest fraction of PA, followed by domestic PA, then travel PA. Leisure-time physical activity was reported to be 0.00 MET·h/week, even at the 75th percentile. We thus calculated levels of participation in LTPA, and these were found to be 7.54% in 2009 and 7.01% in 2015.

Our results showed that the median TC, TG, HDL-C, and LDL-C levels were 4.77 mmol/L, 1.23 mmol/L, 1.39 mmol/L, and 2.91 mmol/L, respectively, in 2009, and 4.89 mmol/L, 1.17 mmol/L, 1.24 mmol/L, and 3.06 mmol/L in 2015, respectively (Appendix A). A significant increase in TC and LDL-C levels and a significant decrease in TG and HDL-C levels were observed. There was a gender difference in serum lipids. The median TC, HDL-C, and LDL-C levels in females were observed to be higher than those in males in each survey, while TG levels in females were statistically significantly lower in the two surveys than those in males.

The overall prevalence of DL was 32.54% in 2009 and 40.53% in 2015 (Appendix A). We observed that the prevalence of hypercholesterolemia and low HDL-C significantly increased between 2009 and 2015. Notably, the overall prevalence of hypertriglyceridemia had a four-percentage-point decrease during the 6 years. In addition, we observed a gender difference in the prevalence of DL subtypes. The prevalence of hypercholesterolemia in women was statistically significantly higher than that in men in 2015, and the prevalence of hypertriglyceridemia and low HDL-C was statistically significantly higher in men than in women in both surveys. The prevalence of combined hyperlipidemia was relatively low, at 3.31% in men and 3.32% in women, respectively. Other characteristics of the samples between the two surveys are listed in Appendix A.

### 3.2. The Distribution of Blood Lipids with Different TPA Levels

**Figure 2:**
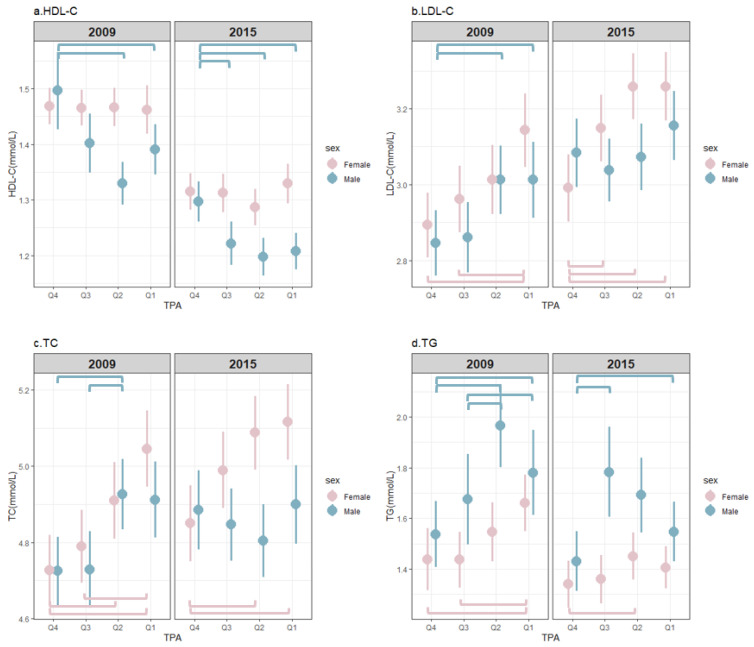
Three-dimensional distribution graph of the serum lipid variables by quartiles of total physical activity among Chinese adults from nine provinces in 2009 and 2015. HDL-C, high-density lipoprotein cholesterol; LDL-C, low-density lipoprotein cholesterol; TC, total cholesterol; TG, hypertriglyceridemia; TPA, total physical activity. Q = quartile. The square bracket indicates the statistically significantly difference between two groups, *p* < 0.05.

Figure 2 presents the distribution of the blood lipids for each gender by quartiles of TPA in 2009 and 2015. The highest TPA level enjoyed the statistically significantly highest concentration of HDL-C in males in both surveys, while there was no significant difference in lipid concentrations among the four TPA levels for females. In 2009, the LDL-C levels in the Q4 group in men were significantly lower than those in the Q1 group and in the Q2 group; however, there was no difference between male groups in 2015. For women, the highest TPA level group had the lowest LDL-C level in both 2009 and 2015. For males, the concentration of TC in the Q4 group and the Q3 group were significantly lower than that in the Q2 group in 2009; however, there were no significant difference in TC levels among the four TPA groups of men in 2015. For females, TC in the Q4 group was observed to be statistically significantly lower than that in the Q1 group and Q2 group, and TC in the Q3 group was also significantly lower than that in the Q1 group in 2009. Total cholesterol in the Q4 group in 2015 was significantly lower than that in the Q1 group and the Q2 group. In 2009, the concentration of TG in males in the Q4 group and the Q3 group was significantly lower than that of the Q1 group and the Q2 group, respectively. Additionally, TG levels in men in the Q4 group in 2015 were significantly lower than those in the Q1 group and the Q3 group. For women, TG levels in both the Q4 group and the Q3 group in 2009 were significantly lower than those in the Q1 group, and in 2015 TG levels in the Q4 group were significantly lower than those in the Q2 group.

**Figure 3:**
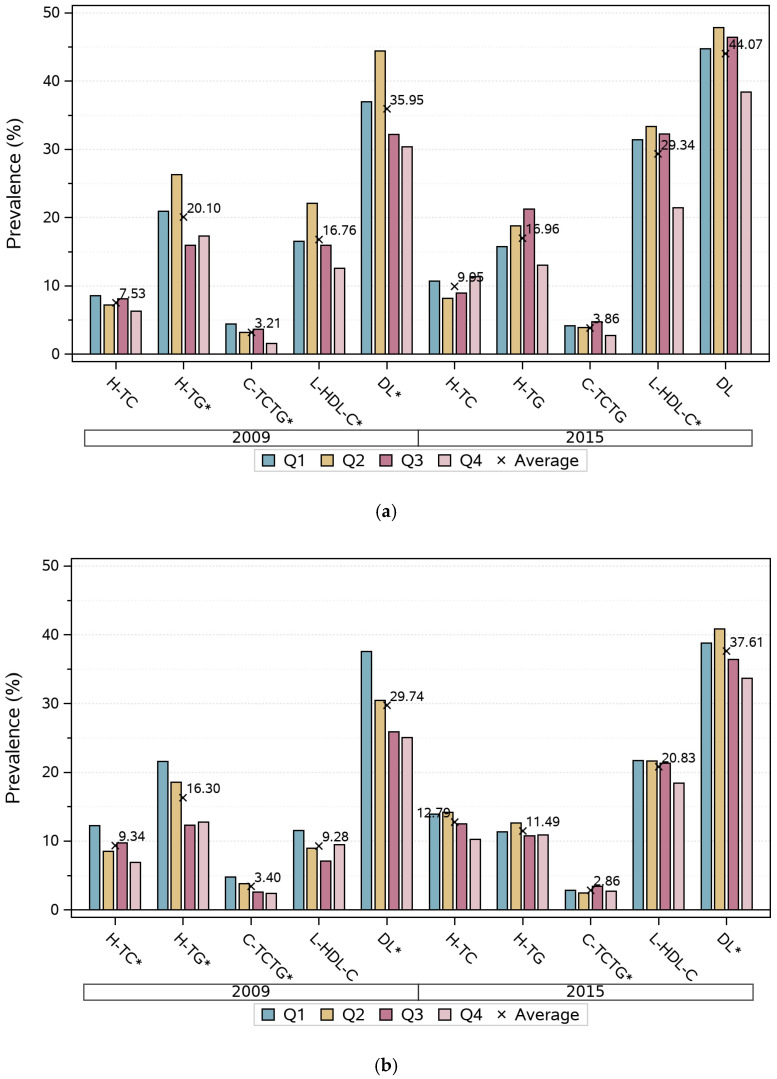
Prevalence of dyslipidemia by quartiles of total physical activity among Chinese adults from nine provinces in 2009 and 2015. H-TC, hypercholesterolemia; H-TG, hypertriglyceridemia; L-HDL-C, low high-density lipoprotein cholesterol; C-TCTG, combined hyperlipidemia; DL, dyslipidemia. Q = quartile. The cross and the number adjacent are the average prevalence of this variable. (**a**) Male; (**b**) Female. Chi-square trend test, * *p* < 0.05.

Figure 3 shows that in 2015 the prevalence of hypercholesterolemia, low HDL-C, and DL was higher than that in 2009. The overall trend for the general prevalence of DL and combined hyperlipidemia stratified by TPA level was an increasing tendency, as the level of TPA decreased. For men, the prevalence of low HDL-C had an increasing trend as TPA declined. Meanwhile, the prevalence of hypertriglyceridemia decreased as TPA levels increased in both genders in 2009; however, these trends were not as obvious in 2015.

### 3.3. Regression Coefficients for Serum Lipid Variables by Quartiles of TPA

Table 2 illustrates the association between TPA levels and the concentration of serum lipids stratified by gender. There are three models for each subgroup. The effect of Model 3 was the best among the three models due to the smallest Akaike information criterion, and contained as many variables related to TC, TG, HDL-C, LDL-C, and DL in previous studies as we considered. Overall, for men, the fourth quartile of TPA significantly increased HDL-C by 0.050 (95% CI = 0.005, 0.096) mmol/L in Model 3, and decreased TG (coefficient equals −0.196 (95% CI = −0.344, −0.049) mmol/L) in Model 1. In women, we found no statistically significant coefficients for any of the serum lipid indices.

### 3.4. Odds Ratio for DL by Quartiles of TPA

**Figure 4:**
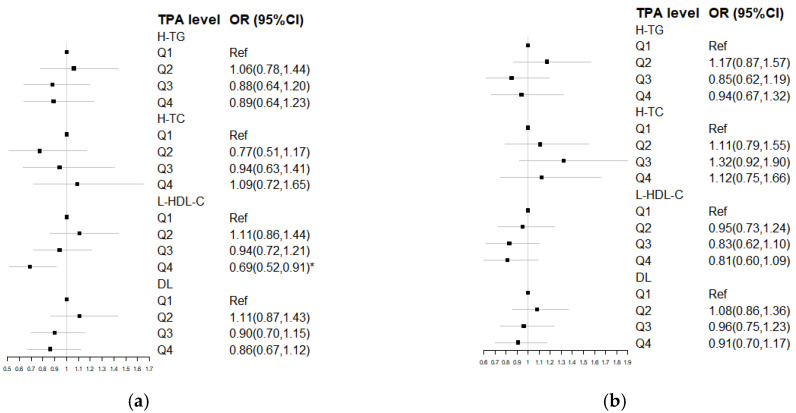
Odds ratios (95% CI) for dyslipidemia by quartiles of total physical activity among Chinese adults from nine provinces. All models used three-level logistic regression adjusted for survey round, baseline age, energy intake, energy from dietary fat, dietary cholesterol, BMI, waist circumference, current smoker, alcohol consumer, education, per capita household income, sedentary activity time, and urbanization index. TPA, total physical activity; H-TG, hypertriglyceridemia; H-TC, hypercholesterolemia; L-HDL-C, low high-density lipoprotein cholesterol; DL, dyslipidemia. Q = quartile. Ref, reference. * *p* < 0.05. (**a**) Male; (**b**) Female.

The forest plots in Figure 4 describe the association between TPA levels and the risk of DL and the DL subtypes. In both genders, the odds-ratios (OR) (95% CI) for the development of DL were found to not be statistically significant. However, in the low HDL-C type in males, the corresponding OR (95% CI) in Model 3 was 0.69 (0.52, 0.91) for participants in the fourth quartile of TPA, compared to that in the lower quartiles. This implies that the risk for developing low HDL-C in the Q4 group was significantly (31%) lower than that in the Q1 group. The ORs (95% CI) for the other subtypes to develop DL were not statistically significant. For females, the ORs (95% CI) between the TPA levels and the three subtypes were calculated to not be statistically significant.

### 3.5. Regression Coefficients for Serum Lipids on Four Types of Physical Activity

Table 3 displays the relationship between four types of PA and serum lipids. The coefficients of occupational PA and HDL-C were observed to be statistically significant both in men and women (0.004 (95% CI = 0.002, 0.007) mmol/L and 0.002 (95% CI = 0.000, 0.004) mmol/L, respectively). The coefficient of travel PA and TG in men was 0.214 (95% CI = 0.020, 0.407) mmol/L. The coefficient of LTPA on TG in men was −0.081 (95% CI = −0.162, −0.001) mmol/L. We found no statistically significant coefficients of domestic PA on any serum lipids.

### 3.6. Regression Coefficients for Serum Lipids on TPA at Baseline Interacted with Change within 6 Years

Table 4 describes the effect of the baseline TPA levels and the changes in blood lipids over the 6-year period. As can be seen, there was a gender difference between TPA and serum lipids. The data show that the effect of TPA on the blood lipids of men was more obvious. For males, compared to participants who had and sustained a low TPA level at baseline with no change over the next six years, participants who increased their TPA over the six-year period would develop a resultant more favorable HDL-C, regardless of the degree of low level TPA. Additionally, the vigorous with stable and decreased groups were also observed to have statistically significant coefficients, which were 0.148 (95% CI = 0.059, 0.238) mmol/L and 0.116 (95% CI = 0.054, 0.178) mmol/L, respectively. While moderate or vigorous basic TPA levels would be expected to also contribute to improvements in LDL-C, TC, and TG, we did not observe statistically significant coefficients in this analysis. For females, the coefficient for the effect of vigorous and increased TPA on HDL-C was calculated to be statistically significant, while this effect was not seen among any other TPA groups and blood lipid indices.

## 4. Discussion

This large-scale prospective survey among participants who were ≥18 years old provides comprehensive estimates of the effects of PA on DL in China. Physical activity should ideally be increased to the highest level to be effective in controlling blood lipids. This implies that as TPA and energy intake drop with aging, a positive association between PA and levels of HDL-C exists (after adjusting for as many reasonable confounders as possible), if the level of PA is high enough. Additionally, occupational PA contributes to higher HDL-C levels, and increasing TPA is beneficial to HDL-C levels in males, no matter what the individual basic level of TPA is. This succinctly explains the association between daily TPA and blood lipids among Chinese adults and contributes to contemporary knowledge related to the precise PA recommendations for each DL subgroup.

The overall prevalence of DL increased from 32.54% to 40.53% between 2009 and 2015 (Appendix A) and was higher in men than in women. The dyslipidemia prevalence reported in past Chinese studies, such as the national chronic kidney disease survey (34.0%) [25] and the China Patient-Centered Evaluative Assessment of Cardiac Events (33.8%) [2], has shown similar results. Countries outside of China, such as Korea (22.3%) [26], Columbia (87.7%) [27], the United Arab Emirates (72.5%) [28], and Iran (73.33%), have reported different and varying DL prevalence rates due to the different racial study populations being investigated, and differing diagnostic criteria used [29]. Specifically, the trends of DL subtypes during the six years are not consistent. Our data showed the increased concentration of LDL-C (from 2.91 mmol/L to 3.06 mmol/L) and TC (from 4.77 mmol/L to 4.89 mmol/L) and the decreased concentration of HDL-C (from 1.39 mmol/L to 1.24 mmol/L) and TG (from 1.23 mmol/L to 1.17 mmol/L) between the two surveys (Appendix A), which means the higher prevalence of hypercholesterolemia (11.50%) and low HDL-C (24.67%) and the lower prevalence of hypertriglyceridemia (13.99%) in 2015 than those in 2009 (8.52%, 12.64%, and 17.99%, respectively).

Even so, we observed participants with the highest TPA level enjoying the lowest prevalence of DL (Figure 3) and the most favorable concentration levels of serum lipids (Figure 2). The likely trend is that as TPA increases, the prevalence of the corresponding DL subtypes seems to decrease. However, the trends were found not to be reliably predictable among all subtypes, and gender differences also exist. In our study, only the highest level of TPA was positively associated with the concentration of HDL-C (by 0.050 mmol/L), and a reduced risk of low HDL-C by 31% in males compared with the lowest one. In previous studies, HDL-C has been observed to be the most likely cholesterol subtype to improve as a result of PA, which concurs with the findings of our study. Mann [13] reported that regular PA has been shown to increase HDL-C, while maintaining and theoretically offsetting increases in LDL-C and TG. There appeared to be a linear dose–response relationship between activity levels and HDL-C levels. However, some other previous studies defined PA as whether the participants regularly met the moderate or vigorous level, which would have resulted in significant associations between PA and the other three lipid profiles. Opoku [30] observed a negative association between regular PA and the risk of DL. Specifically, compared to no regular PA, regular PA decreased the risk of hypercholesterolemia, low HDL-C, high LDL-C, and hypertriglyceridemia by 4%, 5%, 6%, and 11%, respectively. Zhang [31] observed a 11.0% and 10.0% higher risk of lower HDL-C and hypertriglyceridemia, respectively, if participants were classified as physically inactive (where individuals did moderate or vigorous PA for <150 min per week), compared with those classified as physically active (where individuals did moderate or vigorous PA for ≥150 min per week)

High concentrations of serum LDL-C increase the risk of cardiovascular complications. However, HDL-C transports lipids back to the liver for circulation and processing, and thus is protective to the cardiovascular system [32]. Although the precise mechanisms whereby exercise exerts its influence on lipid levels remains unclear, exercise intervention has a significant effect with respect to the improvement of blood lipids [16], especially with long-term aerobic exercise and strength training. The effect of exercise on blood lipid metabolism is influenced by many factors. The regulatory effect of aerobic exercise on lipid metabolism is mainly achieved through its influence on the content and activity of enzymes related to lipid metabolism and receptors, which results in changes in blood lipids [33]. Aerobic exercise improves the ability of skeletal muscle to utilize lipids, and thus lowers blood lipid levels. The possible mechanism underlying this process is likely to be related to the fact that levels of lecithin cholesterol acyltransferase (LCAT) are increased by increased levels of exercise, and this enzyme is responsible for transferring esters to HDL-C, and also increases the activity of lipoprotein lipase [13]. Thus, HDL-C is elevated, along with an increased ability to consume TG [34]. Strength training increases muscle plasma lipoprotein lipase and decreases liver lipoprotein lipase, which leads to a decline in very-low-density lipoprotein cholesterol levels and a rise in HDL-C [35]. After analyzing 51 studies on PA and blood lipids, Kesaniemi [36] pointed out that the most likely PA-induced improvement in the lipid profile is an increase in HDL-C. Lavie [37] observed that targeted strengthening exercises improve HDL-C (mean +6%) and TG (mean −15%), which partially explains the improvement in our study in HDL-C levels in men with increased TPA. Berglund [38] pointed out that long-term high-intensity interval training (HIIT) seemed to be the most appropriate strategy to prevent a decline in HDL-C during a five-year period in men; however, no similar effect of increase in exercise intensity was seen in older women. A reasonable explanation for this could be that muscle mass as a function of proportion of body weight is higher in males than in females. In China, manual labor is mainly performed by males; thus, the improvement in HDL-C in males caused by TPA seems more pronounced than that in females. Additionally, the mediocre contribution of PA to any improvement in HDL-C in females is also likely to be influenced by the menopause. Menopause-induced estrogen deficiency may lead to dysregulated lipid metabolism in females [39], and the overall intensity of PA in menopausal women may not necessarily be high enough to positively influence lipid metabolism.

Triglycerides are measured as part of the routine lipid profile; however, the relationship of TGs to CVD risk has been controversial in the past, and has been overshadowed now by the role of HDL-C [40]. Epidemiological studies have shown that elevated TG levels are independently associated with an increased risk of DL, and are consistent with epidemiologic data showing that coronary heart disease risk increases by 37% (95% CI = 31, 42) per standard deviation (SD) increase in log_e_ TG levels; however, this association is weaker after adjustment for HDL-C levels, and fully compensated for after correction for non-HDL-C [41]. Our data concur with the findings of previous studies; i.e., the effect of TPA on TG appears to be limited according to the results shown in Table 2. The highest TPA level decreased TG by merely −0.196 (95% CI = −0.344, −0.049) mmol/L in Model 1, adjusted for survey round and baseline age.

Our study showed that TPA and the four PA subtypes underwent a dramatic decrease within the six years between 2009 and 2015; even so, occupational PA accounted for the biggest fraction of PA at all times, and LTPA accounted for the smallest fraction of PA, and was calculated to be less than 10% [42]. Specific associations existed between each PA subtype and lipid profiles stratified by gender. Our results indicated that occupational PA (but not LTPA) had the highest weighted role in improving HDL-C in both genders, adjusting for as many confounders as possible. Rural participants accounted for approximately 70% of the participants in the CHNS, and most of their work is done in labor-intensive industries. Therefore, the beneficial effect of occupational PA on HDL-C in these participants is obvious. However, some past evidence implied that different occupational PA levels might lead to differences in association with blood lipid levels [43], and that occupational PA may even lead to a slightly increased risk of CVD [6]. Leisure-time physical activity was only associated with TG levels in men. When LTPA increased by 4 MET·h/week, the concentration of TG decreased by 0.081 mmol/L (95% CI = −0.162, −0.001). Previous studies have observed the beneficial effects of exercise or LTPA on the improvement of serum lipids, especially for HDL-C and TG, similar to the findings of our study [15,16]; however, the benefits do not extend to the entire population due to low rates of LTPA participation and low levels of LTPA in the general population. Therefore, the association between LTPA and lipid profiles seems not to be overtly intimate. As we know, activity benefits general health no matter what type of activities are involved, e.g., doing housework or riding to the office. This is the reason we have focused on TPA as an independent variable, rather than on only one subtype of PA, in the Chinese population study.

Our study shows that a vigorous basic TPA level results in improved HDL-C for men, and that the degree of HDL-C elevation positively correlates with the increase in TPA, no matter what the basic TPA level is in males. The regression coefficient of HDL-C gradually increased in ‘low’, ‘moderate’, and ‘vigorous’ order. At the same basic TPA level, the regression coefficient of HDL-C gradually reduced from ‘increased’, to ‘stable’, and then to ‘decreased’. In one prospective study of 1860 Swedish men, Byberg [44] observed that when 31% of these men increased LTPA, HDL-C levels improved too. However, we have investigated and discussed TPA in our study, and not LTPA or other subtypes of activity, as in previous studies. There are no similar studies in the literature that utilize TPA as a reference. Leskinen [45] conducted a prospective study on 15,634 Finnish participants and found that compared to the high basic LTPA level and an increase over 4 years, those with high basic LTPA levels and a decrease during the same time suffered a 71% higher risk of DL. This implies a higher risk of DL with a negative LTPA change.

Adjustment factors at the time level, the individual level, and the community level were considered in the multilevel models. There are few studies covering as many confounding factors as ours. Body mass index and waist circumference were independently and positively associated with the risk of DL [46,47]. Energy intake, energy from dietary fat, and dietary cholesterol [48] were the variables directly related to blood lipids, and dietary data collection was a feature of CHNS. Although TPA and energy intake decreased together from 2009 to 2015, the relatively high quartile of TPA had a positive effect on HDL-C. The urbanization index [20] represented the third level, which contributed little to blood lipids or dyslipidemia.

Our study extends contemporary knowledge in several important ways. First, our study is one of the largest and most recent studies to show the nationwide characteristics of longitudinal TPA and DL trends in China. Our strict quality control ensured accurate results, which allowed the ability to make certain generalizations. Although the prevalence of DL was consistent with previous studies and meta-analyses, the longitudinal data allowed us to draw robust conclusions with respect to causal relationships. This is the first study discussing the association between TPA, activity subtypes and their change, and DL in China. Studies concerning specific subtypes of PA associated with diseases should be conducted in the future. In addition, to analyze the association between PA and DL, we adjusted for as many confounders as possible, including social demographic characteristics, diet, physical measurements, and urbanization variables, and the estimated values were reasonable and strictly controlled.

Some limitations of this study should be considered. Physical activity was derived from a questionnaire, which may lead to recall bias. However, the questionnaire was broadly acceptable to participants, and was the most economical and useful method to employ for a large-scale survey across nine Chinese provinces. In this 30-year large scale follow-up survey, it is impractical to obtain objective measures of usual activity levels using an accelerometer. The interviewer-administered PA questionnaire concerning frequency, duration, and intensity of PA within 1 week in several different domains has been tested, modified, and used since 1989, and has been derived from the internationally used PA questionnaire. It has been consistently used in each round of the CHNS. Family history and the disease history of DL and medications used were not specially considered and included in the standard questionnaire. To a certain extent, this might cause bias. Third, we excluded a large proportion of the participants from our analysis for varying reasons, and the remaining analytical sample was relatively smaller, thus implying that the generalization of our results to the entire survey population should only be made with caution.

Further study in this area of interest should encompass additional cycles of longitudinal data, and should reveal the PA trajectory for China, and may also reveal more robust associations between the PA trajectory and blood lipids in the Chinese population.

## 5. Conclusions

The findings from the present study indicate that the highest level of TPA is associated with the lowest prevalence of DL. Physical activity is superior in improving blood lipids in males, but not in females. The highest level of TPA is effective in improving the concentration of HDL-C and controlling the risk of low HDL-C in men. Occupational PA contributes to higher HDL-C levels and increasing TPA is beneficial to HDL-C levels in males, even in those with a basic low TPA level. Movement is much more desirable than being sedentary, and LTPA in ordinary people should ideally be raised substantially to improve TPA levels as much as possible in order to be of benefit to prevailing blood lipid levels and DL.

## Figures and Tables

**Table 1:** Demographic characteristics of samples by gender from nine provinces of China in 2009 and 2015.

Characteristics	2009		2015	
	Male (*n* = 1527)	Female (*n* = 1853)	Male (*n* = 1527)	Female (*n* = 1853)
Baseline age (years old)	51.09 (41.72, 59.50)	49.62 (41.48, 58.40)	51.09 (41.72, 59.50)	49.62 (41.48, 58.40)
Married (%)	1378 (90.24)	1679 (90.61)	1401 (91.75)	1654 (89.26) *
Education (%) #	406 (26.59)	324 (17.49) *	478 (31.30)	394 (21.26) *
Annual per capita household income (thousand yuan) #	9.37 (5.15, 15.87)	8.88 (4.72, 15.51)	14.88 (6.65, 27.49)	14.06 (5.74, 26.79)
Urbanization index #	60.32 (50.61, 82.41)	60.11 (50.63, 82.96)	68.82 (55.10, 83.59)	68.57 (55.21, 84.07)
Current smoker (%) #	865 (56.65)	79 (4.26) *	755 (49.44)	54 (2.91) *
Alcohol drinker (%) #	975 (63.85)	172 (9.28) *	847 (55.47)	100 (5.40) *
Energy intake (1000 kcal/day) #	2.39 (2.00, 2.85)	2.00 (1.65, 2.38) *	2.11 (1.69, 2.65)	1.78 (1.43, 2.22) *
Energy from dietary fat (%) #	31.16 (24.27, 38.11)	31.14 (23.98, 38.29)	34.54 (27.18, 43.62)	34.88 (27.15, 43.66)
Dietary cholesterol (mg/d) #	243.36 (137.35, 393.81)	205.92 (104.35, 344.89) *	225.12 (111.64, 376.46)	194.17 (86.20, 327.20) *
BMI (kg/m^2^) #	23.11 (20.87, 25.42)	23.23 (21.20, 25.65)	23.72 (21.36, 26.18)	23.92 (21.80, 26.43) *
Waist circumference (cm) #	84.00 (77.00, 90.10)	81.00 (74.50, 88.00) *	86.00 (79.65, 94.00)	83.00 (76.50, 90.00) *
Sedentary activity time (h) #	16.00 (10.50, 26.00)	14.00 (9.00, 21.00) *	14.00 (9.33, 24.77)	14.00 (7.00, 21.00) *
Participation in LTPA (%)	134 (8.78)	121 (6.53) *	84 (5.50)	153 (8.26) *
TPA (MET·h/w) #	179.20 (80.00, 331.18)	178.51 (81.49, 330.63)	107.67 (29.05, 226.50)	96.68 (45.31, 209.00)
Q4	454.50 (378.00, 625.50)	476.59 (390.58, 613.37)	338.77 (265.00, 452.90)	334.62 (264.24, 475.23)
Q3	244.51 (209.82, 282.62)	247.38 (211.28, 290.30)	156.98 (126.40, 183.75)	152.11 (127.29, 182.93)
Q2	127.75 (104.26, 152.60)	125.68 (104.13, 148.18)	68.97 (52.78, 83.33)	60.83 (49.99, 77.93)
Q1	23.86 (2.63, 48.10)	50.05 (31.56, 64.46)	8.05 (0.00, 24.00)	23.80 (11.96, 32.01)
Occupational PA (MET·h/week) #	152.27 (36.00, 300.00)	96.00 (0.00, 266.00) *	80.00 (0.00, 202.00)	32.00 (0.00, 150.00) *
Travel PA (MET·h/week) #	2.50 (0.00, 6.67)	0.00 (0.00, 6.25) *	0.00 (0.00, 5.00)	0.00 (0.00, 2.50) *
Domestic PA (MET·h/week) #	5.37 (0.00, 24.12)	51.39 (33.02, 75.83) *	7.93 (0.00, 22.52)	36.75 (21.88, 55.88) *
LTPA (MET·h/week)	0.00 (0.00, 0.00)	0.00 (0.00, 0.00) *	0.00 (0.00, 0.00)	0.00 (0.00, 0.00) *
TC (mmol/L) #	4.75 (4.14, 5.39)	4.78 (4.16, 5.43)	4.82 (4.17, 5.43)	4.93 (4.32, 5.63) *
TG (mmol/L) #	1.25 (0.84, 2.02)	1.20 (0.82, 1.77) *	1.20 (0.82, 1.91)	1.15 (0.81, 1.70) *
HDL-C (mmol/L) #	1.33 (1.11, 1.59)	1.44 (1.22, 1.67) *	1.19 (1.00, 1.43)	1.29 (1.07, 1.51) *
LDL-C (mmol/L) #	2.88 (2.31, 3.44)	2.93 (2.38, 3.56) *	3.04 (2.47, 3.62)	3.08 (2.54, 3.72) *
DL (%) #	549 (35.95)	551 (28.74) *	673 (44.07)	697 (37.61) *
Hypercholesterolemia (%) #	115 (7.53)	173 (9.34)	152.00 (9.95)	237.00 (12.79) *
Hypertriglyceridemia (%) #	307 (20.10)	302 (16.30) *	259.00 (16.96)	213.00 (11.49) *
Low HDL-C (%) #	256 (16.76)	172 (9.28) *	448.00 (29.34)	386.00 (20.83) *
Combined hyperlipidemia	49 (3.21)	63 (3.40)	59 (3.86)	53 (2.86)

Marital status, education, current smoker, alcohol consumer, and participation in leisure-time physical activity (LTPA), dyslipidemia (DL), hypercholesterolemia, hypertriglyceridemia, low high-density lipoprotein cholesterol (HDL-C), and combined hyperlipidemia were expressed as number (proportion). Baseline age, annual per capita household income, urbanization index, energy intake, energy from dietary fat, dietary cholesterol, body mass index (BMI), waist circumference, sedentary activity time, total physical activity (TPA), the four subgroups of TPA (occupational PA, travel PA, domestic PA, LTPA), total cholesterol (TC), triglyceride (TG), HDL-C, and LDL-C were expressed as median (25th, 75th) because of their abnormal distribution. Education indicates the proportion of high school and above. Q = quartile; MET = metabolic equivalent of task. Gender difference (* *p* < 0.05). Survey difference (data in Appendix A, # *p* < 0.05).

**Table 2:** Regression coefficients (95% CI) for serum lipid variables by quartiles of total physical activity in a Chinese sample population from nine provinces, CHNS (2009–2015).

Gender	Variables		Q1	Q2	Q3	Q4
Male	HDL-C (mmol/L)	Model 1	Ref	−0.020 (−0.064, 0.023)	0.033 (−0.010, 0.077)	0.080 (0.036, 0.125) *
		Model 2	Ref	−0.021 (−0.064, 0.023)	0.022 (−0.021, 0.065)	0.060 (0.016, 0.105) *
		Model 3	Ref	−0.023 (−0.066, 0.021)	0.017 (−0.027, 0.060)	0.050 (0.005, 0.096) *
	LDL-C (mmol/L)	Model 1	Ref	−0.015 (−0.098, 0.068)	−0.055 (−0.138, 0.028)	−0.027 (−0.113, 0.059)
		Model 2	Ref	−0.014 (−0.097, 0.070)	−0.039 (−0.123, 0.045)	−0.005 (−0.092, 0.083)
		Model 3	Ref	−0.012 (−0.095, 0.071)	−0.035 (−0.118, 0.049)	0.003 (−0.085, 0.092)
	TC (mmol/L)	Model 1	Ref	0.012 (−0.076, 0.099)	−0.040 (−0.128, 0.048)	0.004 (−0.087, 0.095)
		Model 2	Ref	0.004 (−0.085, 0.092)	−0.036 (−0.125, 0.053)	0.016 (−0.077, 0.109)
		Model 3	Ref	0.006 (−0.082, 0.095)	−0.029 (−0.118, 0.060)	0.028 (−0.066, 0.122)
	TG (mmol/L)	Model 1	Ref	0.051 (−0.097, 0.198)	−0.054 (−0.200, 0.092)	−0.196 (−0.344, −0.049) *
		Model 2	Ref	0.029 (−0.116, 0.174)	−0.040 (−0.184, 0.104)	−0.145 (−0.292, 0.002)
		Model 3	Ref	0.038 (−0.107, 0.183)	−0.016 (−0.161, 0.129)	−0.103 (−0.252, 0.047)
Female	HDL-C (mmol/L)	Model 1	Ref	−0.013 (−0.040, 0.014)	−0.004 (−0.032, 0.024)	0.014 (−0.015, 0.044)
		Model 2	Ref	−0.014 (−0.041, 0.013)	0.001 (−0.028, 0.030)	0.018 (−0.012, 0.048)
		Model 3	Ref	−0.014 (−0.041, 0.013)	0.002 (−0.027, 0.031)	0.020 (−0.010, 0.050)
	LDL-C (mmol/L)	Model 1	Ref	0.012 (−0.054, 0.077)	0.010 (−0.059, 0.079)	−0.065 (−0.138, 0.007)
		Model 2	Ref	0.027 (−0.039, 0.094)	0.024 (−0.046, 0.094)	−0.045 (−0.119, 0.029)
		Model 3	Ref	0.028 (−0.038, 0.095)	0.028 (−0.043, 0.098)	−0.038 (−0.113, 0.037)
	TC (mmol/L)	Model 1	Ref	0.016 (−0.055, 0.087)	0.013 (−0.062, 0.088)	−0.042 (−0.121, 0.037)
		Model 2	Ref	0.038 (−0.035, 0.110)	0.035 (−0.041, 0.112)	−0.010 (−0.091, 0.071)
		Model 3	Ref	0.039 (−0.033, 0.111)	0.041 (−0.036, 0.118)	0.000 (−0.081, 0.082)
	TG (mmol/L)	Model 1	Ref	0.013 (−0.072, 0.099)	−0.023 (−0.114, 0.067)	−0.049 (−0.142, 0.045)
		Model 2	Ref	0.030 (−0.055, 0.115)	−0.018 (−0.107, 0.072)	−0.035 (−0.128, 0.059)
		Model 3	Ref	0.031 (−0.054, 0.116)	−0.016 (−0.106, 0.074)	−0.031 (−0.127, 0.064)

All the models were constructed using three-level random intercept growth models. Model 1 adjusted total physical activity, survey round, baseline age; Model 2 adjusted Model 1 + energy intake, energy for dietary fat, dietary cholesterol, BMI, waist circumference, current smoker, alcohol drinker, education, per capita household income, sedentary activity time; Model 3 adjusted Model2 + urbanization index. HDL-C, high-density lipoprotein cholesterol; LDL-C, low-density lipoprotein cholesterol; TC, total cholesterol; TG, hypertriglyceridemia; PA, physical activity; LTPA, leisure-time physical activity. Q = quartile. Ref, reference. * *p* < 0.05.

**Table 3:** Regression coefficients (95% CI) for serum lipids on the four subtypes of physical activity in Chinese adults from nine provinces, CHNS (2009–2015).

Variables	Occupational PA	Travel PA	Domestic PA	LTPA
Male				
HDL-C (mmol/L)	0.004 (0.002, 0.007) *	−0.057 (−0.115, 0.001)	−0.008 (−0.023, 0.006)	0.007 (−0.017, 0.031)
LDL-C (mmol/L)	0.003 (−0.002, 0.008)	0.022 (−0.089, 0.133)	0.012 (−0.016, 0.040)	0.007 (−0.040, 0.054)
TC (mmol/L)	0.005 (−0.000, 0.010)	0.044 (−0.073, 0.162)	−0.015 (−0.045, 0.015)	−0.009 (−0.059, 0.040)
TG (mmol/L)	−0.006 (−0.014, 0.003)	0.213 (0.020, 0.407) *	−0.029 (−0.078, 0.019)	−0.081 (−0.162, −0.001) *
Female				
HDL-C (mmol/L)	0.002 (0.000, 0.004) *	−0.010 (−0.047, 0.027)	−0.004 (−0.009, 0.002)	0.004 (−0.013, 0.021)
LDL-C (mmol/L)	−0.003 (−0.007, 0.002)	−0.002 (−0.093, 0.089)	−0.009 (−0.022, 0.004)	−0.020 (−0.062, 0.023)
TC (mmol/L)	−0.001 (−0.005, 0.004)	−0.034 (−0.133, 0.066)	−0.002 (−0.016, 0.013)	−0.023 (−0.069, 0.023)
TG (mmol/L)	−0.004 (−0.009, 0.002)	−0.066 (−0.183, 0.051)	0.008 (−0.009, 0.024)	−0.019 (−0.073, 0.035)

All the models were constructed using three-level random intercept growth models, adjusting for survey round, baseline age, energy intake, energy from dietary fat, dietary cholesterol, BMI, waist circumference, current smoker, alcohol consumer, education, per capita household income, sedentary activity time, and urbanization index. HDL-C, high-density lipoprotein cholesterol; LDL-C, low-density lipoprotein cholesterol; TC, total cholesterol; TG, hypertriglyceridemia; PA, physical activity; LTPA, leisure-time physical activity. Q = quartile. * *p* < 0.05.

**Table 4:** Regression coefficients (95% CI) for serum lipids on total physical activity at baseline coupled with the change within six years among Chinese adults from nine provinces, CHNS (2009–2015).

TPA Levels and Change	HDL-C (mmol/L)	LDL-C (mmol/L)	TC (mmol/L)	TG (mmol/L)
Male				
Vigorous and increased	0.197 (0.101, 0.293) *	−0.090 (−0.294, 0.114)	0.005 (−0.220, 0.230)	−0.216 (−0.536, 0.104)
Vigorous and stable	0.148 (0.059, 0.238) *	−0.147 (−0.335, 0.040)	−0.170 (−0.378, 0.038)	−0.526 (−0.825, −0.226) *
Vigorous and decreased	0.116 (0.054, 0.178) *	−0.137 (−0.268, −0.005) *	−0.149 (−0.294, −0.003) *	−0.401 (−0.603, −0.199) *
Moderate and increased	0.114 (0.043, 0.185) *	−0.113 (−0.263, 0.038)	−0.078 (−0.244, 0.089)	−0.264 (−0.502, −0.026) *
Moderate and stable	0.033 (−0.036, 0.101)	−0.066 (−0.210, 0.077)	−0.088 (−0.247, 0.071)	−0.136 (−0.366, 0.094)
Moderate and decreased	0.029 (−0.044, 0.102)	−0.172 (−0.325, −0.018) *	−0.229 (−0.399, −0.059) *	−0.233 (−0.480, 0.014)
Low and increased	0.075 (0.014, 0.137) *	−0.095 (−0.224, 0.033)	−0.074 (−0.217, 0.069)	−0.133 (−0.341, 0.075)
Female				
Vigorous and increased	0.094 (0.014, 0.175) *	−0.009 (−0.213, 0.195)	0.031 (−0.192, 0.254)	−0.194 (−0.424, 0.036)
Vigorous and stable	0.023 (−0.052, 0.097)	0.048 (−0.141, 0.238)	0.064 (−0.142, 0.271)	−0.041 (−0.255, 0.173)
Vigorous and decreased	0.019 (−0.027, 0.064)	−0.041 (−0.158, 0.077)	−0.040 (−0.168, 0.088)	−0.088 (−0.218, 0.042)
Moderate and increased	0.032 (−0.023, 0.086)	−0.065 (−0.202, 0.072)	−0.047 (−0.197, 0.102)	0.003 (−0.153, 0.158)
Moderate and stable	0.032 (−0.019, 0.082)	0.086 (−0.042, 0.213)	0.130 (−0.009, 0.269)	−0.016 (−0.161, 0.130)
Moderate and decreased	0.030 (−0.027, 0.087)	−0.008 (−0.152, 0.137)	0.007 (−0.150, 0.165)	−0.069 (−0.233, 0.095)
Low and increased	0.0260 (−0.021, 0.073)	−0.025 (−0.142, 0.093)	0.000 (−0.128, 0.129)	0.002 (−0.132, 0.136)

All the models were constructed using three-level random intercept growth models, adjusted for basic total physical activity (TPA) level × change in TPA in 6 years, survey round, baseline age, energy intake, energy from dietary fat, dietary cholesterol, BMI, waist circumference, current smoker, alcohol consumer, and sedentary activity time. HDL-C, high-density lipoprotein cholesterol; LDL-C, low-density lipoprotein cholesterol; TC, total cholesterol; TG, hypertriglyceridemia. * *p* < 0.05.

## Data Availability

The datasets generated during and/or analyzed during the present study are available from the author (B.Z.) upon reasonable request.

## Appendix A

**Table A1:** Demographic characteristics of samples from nine provinces of China in 2009 and 2015.

Characteristics	2009	2015
Baseline age (years old)	50.52 (41.57, 58.87)	50.52 (41.57, 58.87)
Married (%)	3057 (90.44)	3055 (90.38)
Education (%) #	730 (21.60)	872 (25.80)
Annual per capita household income (thousand yuan) #	9.16 (4.89, 15.73)	14.42 (6.15, 27.13)
Urbanization index #	14.00 (9.83, 23.33)	14.00 (7.50, 22.17)
Current smoker (%) #	944 (27.93)	809 (23.93)
Alcohol drinker (%) #	1147 (33.93)	947 (28.02)
Energy intake (1000 kcal/day) #	2.16 (1.78, 2.63)	1.93 (1.52, 2.42)
Energy from dietary fat (%) #	31.15 (24.12, 38.22)	34.71 (27.18, 43.64)
Dietary cholesterol (mg/d) #	221.02 (119.13, 367.34)	207.26 (99.27, 350.34)
BMI (kg/m^2^) #	23.19 (21.06, 25.56)	23.82 (21.63, 26.30)
Waist circumference (cm) #	82.00 (75.50, 89.50)	85.00 (78.00, 92.00)
Sedentary activity time (h) #	14.00 (9.83, 23.33)	14.00 (7.50, 22.17)
Participation in LTPA (%)	255 (7.54)	237 (7.01)
TPA (MET·h/w) #	178.74 (80.81, 330.90)	100.49 (39.67, 216.50)
Q4	466.84 (384.87, 617.61)	337.06 (264.38, 462.5)
Q3	245.12 (210.00, 287.00)	153.6 (127.04, 183.28)
Q2	126.52 (104.20, 149.48)	62.86 (51.28, 80.34)
Q1	40.78 (16.10, 59.13)	17.21 (0, 28.88)
Occupational PA (MET·h/week) #	120.00 (0.00, 280.00)	55 (0, 175)
Travel PA (MET·h/week) #	1.25 (0.00, 6.67)	0.00 (0, 3.75)
Domestic PA (MET·h/week) #	32.36 (5.37, 58.71)	23.8 (5.37, 43.75)
LTPA (MET·h/week)	0.00 (0.00, 0.00)	0.00 (0.00, 0.00)
TC (mmol/L) #	4.77 (4.15, 5.42)	4.89 (4.25, 5.53)
TG (mmol/L) #	1.23 (0.83, 1.88)	1.17 (0.81, 1.77)
HDL-C (mmol/L) #	1.39 (1.17, 1.64)	1.24 (1.04, 1.47)
LDL-C (mmol/L) #	2.91 (2.35, 3.50)	3.06 (2.51, 3.68)
DL (%) #	1100 (32.54)	1370 (40.53)
Hypercholesterolemia (%) #	288 (8.52)	388.00 (11.50)
Hypertriglyceridemia (%) #	608 (17.99)	472.00 (13.99)
Low HDL-C (%) #	427 (12.64)	832.00 (24.67)
Combined hyperlipidemia	112 (3.31)	112 (3.32)

Marital status, education, current smoker, alcohol drinker, and participation in leisure-time physical activity (LTPA), dyslipidemia (DL), hypercholesterolemia, hypertriglyceridemia, low high-density lipoprotein cholesterol (HDL-C) and combined hyperlipidemia were expressed as number (proportion). Baseline age, annual per capita household income, urbanization index, energy intake, energy from dietary fat, dietary cholesterol, body mass index (BMI), waist circumference, sedentary activity time, total physical activity (TPA), the four subgroups of TPA, occupational PA, travel PA, domestic PA, LTPA, total cholesterol (TC), triglyceride (TG), HDL-C, and LDL-C were expressed as median (25th, 75th) because of the abnormal distribution. Education indicated the proportion of high school and above. Q = quartile; MET = metabolic equivalent of task. Survey difference, # *p* < 0.05.
